# The Multifaceted Role of Human Dickkopf-3 (DKK-3) in Development, Immune Modulation and Cancer

**DOI:** 10.3390/cells13010075

**Published:** 2023-12-29

**Authors:** Jana Mourtada, Chloé Thibaudeau, Bohdan Wasylyk, Alain C. Jung

**Affiliations:** 1 Laboratoire de Biologie Tumorale, Institut de Cancérologie Strasbourg Europe, 67200 Strasbourg, France; jana.mourtada@net.usj.edu.lb (J.M.); chloe.thibaudeau@etu.unistra.fr (C.T.); 2 Laboratoire STREINTH (Stress Response and Innovative Therapies), INSERM U1113 IRFAC, Université de Strasbourg, 67200 Strasbourg, France; 3 Institut de Génétique et de Biologie Moléculaire et Cellulaire (IGBMC), 67404 Illkirch Graffenstaden, France; boh@igbmc.fr; 4 Institut National de la Santé et de la Recherche Médicale (INSERM), U 1258, 67404 Illkirch Graffenstaden, France; 5 Centre Nationale de la Recherche Scientifique (CNRS), UMR 7104, 67404 Illkirch Graffenstaden, France; 6 Université de Strasbourg, 67000 Strasbourg, France

**Keywords:** Dickkopf (DKK) family, Wnt signaling pathway, DKK-3, development, immunomodulation, cancer, tumor suppressor, oncogene, therapy

## Abstract

The human Dickkopf (DKK) family includes four main secreted proteins, DKK-1, DKK-2, DKK-3, and DKK-4, as well as the DKK-3 related protein soggy (Sgy-1 or DKKL1). These glycoproteins play crucial roles in various biological processes, and especially modulation of the Wnt signaling pathway. DKK-3 is distinct, with its multifaceted roles in development, stem cell differentiation and tissue homeostasis. Intriguingly, DKK-3 appears to have immunomodulatory functions and a complex role in cancer, acting as either a tumor suppressor or an oncogene, depending on the context. DKK-3 is a promising diagnostic and therapeutic target that can be modulated by epigenetic reactivation, gene therapy and DKK-3-blocking agents. However, further research is needed to optimize DKK-3-based therapies. In this review, we comprehensively describe the known functions of DKK-3 and highlight the importance of context in understanding and exploiting its roles in health and disease.

## 1. Introduction

Embryonic development involves various processes, including cell proliferation, fate determination, differentiation, migration and axis patterning. These processes are shut down in most cells in the adult, except for stem cells, which use them for tissue homeostasis and maintenance [1]. They are regulated by signaling pathways that include Wnt, Notch and Hedgehog (for review, see [2] and references therein). Uncontrolled reactivation of these pathways during adult life, through the acquisition of somatic genetic mutations or epigenetic anomalies, are at the origin of malignant transformation, tumor progression and cancer cell resistance to therapy [2].

Inhibitors of the Wnt signaling pathway are being tested in clinical trials [3]. However, there are other promising avenues to explore, especially regulation by one particular extracellularly secreted factor, DKK-3. The various extracellularly secreted regulators include the Wnt inhibitory factor-1 (WIF-1), soluble Frizzled-related proteins (sFRPs), Cerberus, R-spondin and the Dickkopf (DKK) family [4]. There are four DKK factors that are classically known to downregulate Wnt signaling through interactions with the co-receptor LRP5/6 and that have additional, context-dependent functions. The goal of this review is to briefly summarize the role of DKK proteins in the regulation of the canonical Wnt signaling pathway and to further focus on the intriguing complex functions of the most divergent member, DKK-3, which functions as both an oncogene and a tumor suppressor, and which has promise as either a prognostic biomarker or a therapeutic target in human cancers.

## 2. Sequence and Structural Properties of the DKK Family of Proteins

**Figure 1:**
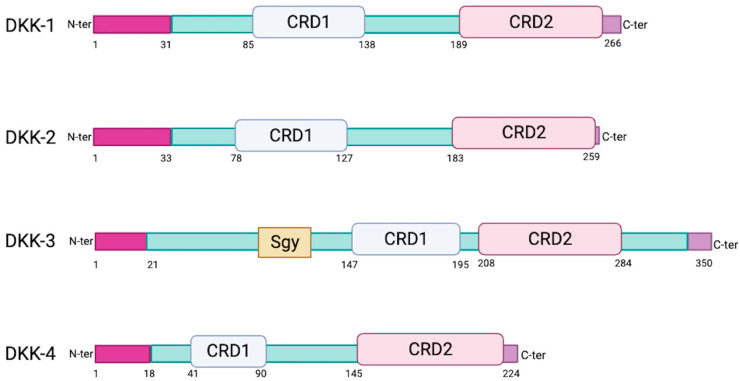
Schematic diagrams of individual DKK proteins. CRD1/CRD2: cysteine-rich domain 1 or 2. Sgy: Soggy. Created with BioRender.com (accessed on 11 November 2023).

The human genome encodes four similar secreted DKK proteins (DKK-1, DKK-2, DKK-3 and DKK-4) (Figure 1) and a distinct DKK-3-related protein known as Soggy (Sgy-1 or DKKL1) [5,6]. DKK proteins are glycosylated and harbor an N-terminal signal peptide as well as two conserved cysteine-rich domains (CRD1 and CRD2). CRD1 is located towards the N-terminus and is uniquely found in the human DKK family of proteins [7]. Both CRD1 and CRD2 are required for receptor binding at the cell surface (see below). DKK-3 is the most divergent family member. It has a shorter linker between its CRD1 and CRD2 (13 amino acids versus 51 to 56 for the others), lower homology (40% versus 50% between the others) [8,9], a higher molecular weight (38 kDa compared to 24 to 29 kDa [9]), and an additional Soggy domain [10] that resembles the N-terminal region of DKKL1. The functional significance of the Soggy domain remains unknown.

## 3. DKKs Modulate the Wnt Signaling Pathway through Binding to the LRP5/6 and Krm1/2 Co-Receptors

DKK-1 was initially discovered in Xenopus embryos as a crucial regulator of the Wnt/β-catenin signaling pathway, early embryonic development and head formation [11]. Wnt signaling includes three sub-pathways, namely the canonical as well as the non-canonical planar cell polarity (PCP) and Wnt/calcium pathways [12,13,14].

**Figure 2:**
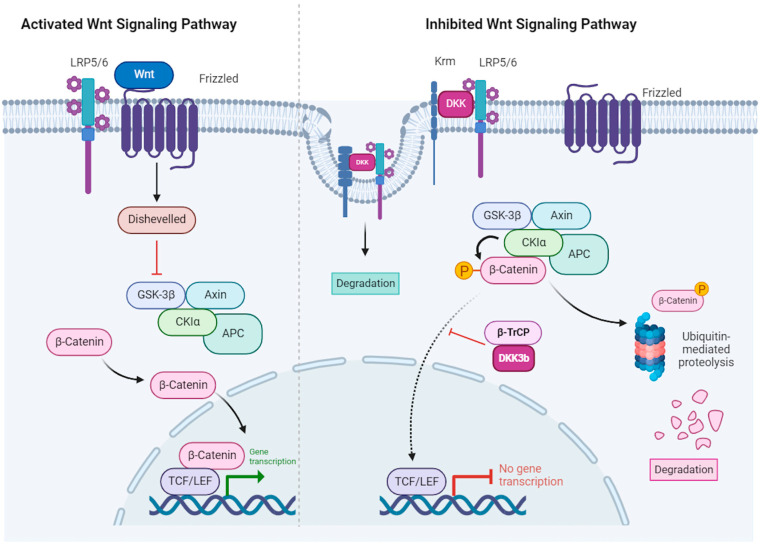
Canonical Wnt signaling in the presence of Wnt ligands (**left** panel) and inhibition by DKK (**right** panel). Left panel: Wnt binds to a receptor complex on the cell surface that is composed of Frizzled (FZD) and either lipoprotein receptor-related protein 5 (LRP5) or LRP6. This interaction leads to activation of Dishevelled (DVL) that then inhibits the Axin–glycogen synthase kinase 3β (GSK3β)-adenomatous polyposis coli (APC)-casein kinase 1 (CK1) complex, leading to loss of β-catenin phosphorylation and degradation. β-catenin accumulates in the cytoplasm and translocates to the nucleus, where it activates the transcription factors T-cell factor (TCF) and lymphoid enhancer factor (LEF). Right panel: Dickkopf (DKK) inhibits Wnt–β-catenin signaling. DKK binds to LRP5/6, preventing its interaction with FZD. Additionally, it collaborates with Kremen (Krm) to trigger internalization and degradation of the LRP6-DKK complex. Finally, the protein complex (DVL-GSK3β-APC-CK1-Axin) facilitated the phosphorylation of β-catenin leading to its ubiquitinylation and degradation by the proteasome (adapted from Schunk et al., 2021 [17]). Canonical Wnt signaling inhibition by diffusible DKKs is brought about by their binding to the LRP5/6 FZD co-receptor. It has been proposed that extracellular DKK-3 triggers LRP5/6 internalization and degradation via binding to Kremen1/2. The non-secreted DKK-3b isoform was shown to interact with the ß-TrCP ubiquitin–ligase and impair ß-catenin translocation. Created with BioRender.com (accessed on 18 December 2023).

In the canonical pathway, Wnt regulates the stability and cellular localization of β-catenin. In the absence of Wnt signaling (Figure 2, right panel), β-catenin is degraded through a sequence of events that involves: (1) binding to a multi-protein complex that contains Axin, the glycogen synthase kinase 3β (GSK 3β) and adenomatous polyposis coli (APC), (2) phosphorylation by GSK 3β and CK1α, (3) ubiquitination by the β-transducin repeats-containing proteins (β-TrCP) E3-ubiquitin ligase (not shown) and (4) degradation by the proteasome. In the presence of Wnt (Figure 2, left panel), β-catenin is stabilized by: (1) Wnt binding to the Frizzled receptors and the LRP5/6 (low-density lipoprotein receptor-related protein 5/6) co-receptors (2) phosphorylation of disheveled leading to inhibition of the Axin/GSK 3β/APC complex, (3) accumulation of unphosphorylated β-catenin that is resistant to ubiquitination and degradation, (4) β-catenin translocation to the nucleus, association with the T-cell factor/lymphoid enhancer factor (TCF/LEF) transcription factors and regulation of Wnt target genes [15,16].

Canonical Wnt signaling is inhibited by DKK-1/-2 and -4 binding to the Wnt co-receptors LRP5/6 [1,4,18] (Figure 2, right panel). LRP5/6 were initially discovered as plasma membrane receptors for DKK-1 and DKK-2, and LRP6 has been shown to be a co-receptor for Wnt signaling. Mao et al. originally observed that DKK-1 binds specifically and with high affinity (10^−10^ M) to LRP6 expressed on 293T cells, and inhibits the Wnt pathway by impeding the formation of the Wnt–Frizzled–LRP complex [19]. DKKs also inhibit Wnt signaling by binding with high affinity to Kremen proteins (Krm1 and Krm2), which results in the formation of ternary complexes with LRP5/6, rapid endocytosis and degradation of LRP5/6 [20] (Figure 2, right panel). Consistently, mice with a double knockout of Krm1 and Krm2 (Krm1/1 and Krm2/2) have elevated Wnt signaling, a bone mass phenotype and extra digits in the forelimbs. Notably, the growth of these ectopic digits is further amplified by the loss of DKK expression, indicating that DKK-1 and Kremen interact genetically during limb development [21].

DKK-3 differs from the other DKKs in the way it impinges on the Wnt signaling pathway. However, there are discordant findings. Nierhs and collaborators reported that DKK-3 does not bind to LRP6 or Kremen-2 co-receptors at the cell surface, consistent with the lack in its CRD2 of specific amino acids required for protein–protein interactions [22]. Similarly, Mao et al. reported that DKK-3 does not inhibit Wnt induction of transcription in 293T cells, suggesting that DKK-3 does not antagonize Wnt signaling [19]. Yet, several studies have reported that DKK-3 inhibits the Wnt signaling pathway by interacting with Kremen receptors. Mohammadpour et al. predicted, using bioinformatics models for docking analysis, that DKK-3 interacts with either or both the extracellular kringle and CUB domains of Kremen [23]. They proposed that the interaction with the CUB domain potentiates the Wnt pathway, leading to enhanced cell invasion and migration, whereas the interaction with either the kringle domain or both the kringle and CUB domains prevents Wnt signaling by inhibiting the nuclear translocation of β-catenin [24]. Xu et al. reported that DKK-3 and Kremen-1 interact functionally [25]. Downregulation of Kremen-1 with siRNA reduced the protective ability of rDKK-3 in a mouse intracerebral hemorrhage (ICH) model. In addition, DKK-3 and Kremen-1 co-localized in neurons and microglia after ICH [25]. Ferrari et al. [26] reported that DKK-3 interacts physically with Kremen-1 in human breast cancer–cancer-associated fibroblasts (BC-CAFs) and co-localizes in internal structures, leading to Kremen-1 destabilization, LRP5/6 stabilization and activation of Wnt signaling in a cell-autonomous manner.

There is also a cytoplasmic, non-secreted isoform, DKK-3b. It was initially identified as the 29-kDa subunit of the short-lived, membrane-bound enzyme, type 2 deiodinase (D2p29), that displays intracellular trafficking in a myosin-5-dependent manner in rat astrocytes [27,28]. It is encoded by an alternative transcript that starts in intron 2 of the DKK-3 gene. DKK-3b lacks the N-terminal 71 residues, which include the signal sequence and N-glycosylation sites that direct DKK-3 to the secretory vesicle 22 (for review see [29] and references therein). DKK-3b is an intracellular regulator of β-catenin signaling and cell proliferation [30]. It impairs the nuclear translocation of β-catenin through the formation of an extranuclear complex with β-TrCP, thereby sequestering cytoplasmic, unphosphorylated β-catenin, and inhibiting Wnt/β-catenin signaling (Figure 2, right panel).

In conclusion, DKK-3′s properties are contentious and different from the other DKK family members. Due to the controversies about its ability to physically bind LRP5/6 and/or Kremen 1/2 at the cell surface, it was considered by some authors to be an orphan ligand until the late 2010s.

## 4. CKAP4 Is the Only Known Receptor for DKK-3

Quite recently, Kimura et al. [31] discovered that DKK-3, as well as the other DKKs, physically bind to CKAP4 (cytoskeleton-associated protein 4, also known as P63, CLIMP-63 or ERGIC-63). CKAP4 is a type II transmembrane protein that is primarily expressed in the endoplasmic reticulum (ER) and regulates its architecture [32]. Yet, a minor fraction of CKAP4 (less than 10% of the total) is transported to the plasma membrane of specific cell types, including pulmonary, vascular smooth muscle, and bladder epithelial cells [33]. CKAP4 is the only known protein that binds to the CRD1 domain [31], whereas LRP5/6 binds to CRD2 [1,4,18]. The CKAP4 extracellular domain–CRD1 interaction triggers the internalization of CKAP4 in a clathrin-dependent manner [31]. The functional consequences of DKK binding to CKAP4 on the cell membrane are poorly understood. Most notably, DKK-1 binding to CKAP4 triggers the intracellular PI3K/Akt signaling pathway [31], which is relevant for tumor progression (for review see [34] and references therein). To date, and to the best of our knowledge, CKAP4 is the only known receptor for DKK-3.

## 5. DKK-3 Has Diverse Biological Roles

**Figure 3:**
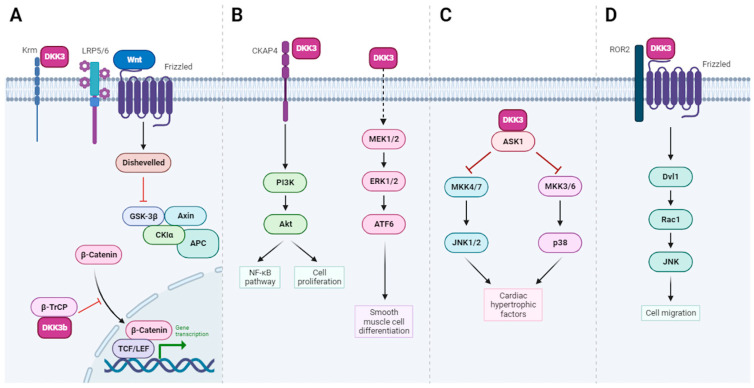
Pathways regulated by DKK-3: insights into DKK-3 functions. (**A**). In addition to signaling by extracellular DKK-3 (see also Figure 2), the DKK-3b variant serves as an intracellular regulator of β-catenin signaling and cell proliferation. DKK-3b, in complex with β-TrCP, impedes the nuclear translocation of β-catenin, leading to the cytoplasmic sequestration, unphosphorylated β-catenin and subsequent inhibition of Wnt/β-catenin signaling. (**B**). Left pathway: Secreted DKK-3 activates NF-κB signaling via a CKAP4-Akt-NF-κB axis. Right pathway: extracellular DKK-3 promotes the differentiation of stem cells into vascular smooth muscle cells through the activation of the MEK/ERK/ATF6 axis. (**C**). Intracellular DKK-3 prevents cardiac hypertrophy through ASK1/JNK/p38 signaling pathway activation. (**D**). Extracellular DKK-3 stimulates the migration of HUVEC endothelial cells through the Wnt/planar cell polarity (ROR2-Dvl1-Rac1-JNK) signaling pathway. Created with BioRender.com (accessed on 18 December 2023).

Unlike other DKK members, DKK-3 has no major developmental function, and DKK-3 knock-out mice are viable and fertile. They display different phenotypes that include behavior (increased velocity), elevated hematocrit and total hemoglobin concentration in blood, lower respiratory rates and higher titers of NK cells and IgM [35]. Intriguingly, the specific knock-out of DKK-3b is embryonically lethal [30]. Leonard and collaborators engineered mouse models with a specific knockdown of the DKK-3b transcript, using a promoter trap knock-in strategy. DKK-3b loss in mouse embryonic fibroblasts results in elevated Wnt–ß-catenin signaling [30] (Figure 3A). More recently, DKK-3b has been shown to be involved in the cell-autonomous regeneration of pancreatic ß-cells and cardiomyocytes in Zebrafish [36,37]. How these functions of DKK-3b are relevant to human biology remains to be determined.

DKK-3 has additional roles in humans, relating to cartilage degradation, cardiac hypertrophy, artheroprotection, pulmonary ventilation and oxidative stress (Cell protection, Table 1).

Snelling et al. demonstrated that incubation of primary human chondrocytes or chondrosarcoma cells with recombinant DKK-3 protects against in vitro cartilage degradation through enhanced TGF-β signaling, and DKK-3 expression is regulated by both injury and inflammatory cytokines [39]. Intracellular and extracellular DKK-3 play crucial roles in preventing cardiac hypertrophy [63] and promote the differentiation of stem cells into vascular smooth muscle cells [41], via the activation of several intracellular signaling pathways, including the ASK1/JNK/p38 [apoptosis signal-regulating kinase 1 (ASK1)-c-Jun N-terminal kinase (JNK)/p38] and MEK/ERK/ATF6 axes, respectively (Figure 3B,C).

Extracellular DKK-3 also serves as an atheroprotective cytokine, which stimulates the migration of HUVEC endothelial cells through the Wnt/planar cell polarity (ROR2-Dvl1-Rac1-JNK) signaling pathway, potentially acting as a biomarker of endothelial integrity and repair [42] (Figure 3D). DKK-3 can also influence cellular antioxidant defense mechanisms and protect cells from oxidative damage since recombinant DKK-3 protects cultured astrocytes against oxidative stress [43]. However, in all these cases, the precise molecular mechanisms have not been elucidated.

## 6. DKK-3 Modulates the Immune System

DKK-3 has a diverse role in immunomodulation that extends from differentiation of B-cells, immune peripheric tolerance, dendritic cell differentiation and inflammation (Table 1). DKK-3 is expressed at the highest levels in immune-privileged organs, such as the embryo, placenta, eye and brain [9], which is compatible with a role for DKK-3 in their immune tolerance. Ludwig et al. [44] provided evidence that DKK-3 acts as a modulator of B-cell fate and function. They found that loss of DKK-3 in *DKK-3^−/−^* mice had significant impacts on various aspects of B-cell biology. Specifically, it affected the maturation of B2 cells, which play a crucial role in adaptive immune responses. It also reduced the proliferation and self-maintenance ability of B1 cells in the periphery, thereby influencing the production of antibodies and cytokines involved in the innate immune response. Most importantly, DKK-3 has been shown to play a vital role in establishing peripheral CD8 T-cell tolerance in a T-cell receptor (TCR) transgenic system. This is supported by the observation that DKK-3 expression is elevated in tolerant CD8 T-cells, and it contributes to reducing the overall reactivity of CD8 T-cells in vitro [45]. Interestingly, the same group also reported that an anti-DKK-3 neutralizing antibody, administered after transplantation of male MHC class-I mismatched embryoid bodies under the kidney capsule of sex-matched recipient mice, resulted in a higher occurrence of graft rejection. This suggests that DKK-3 is involved in the local microenvironment that protects transplanted MHC class-I mismatched embryo bodies from CD8+ T-cell-dependent rejection. In addition, they provided evidence that DKK-3 plays a role in regulating CD4+ T-cell-mediated autoimmune encephalomyelitis, by restricting T-cell activation and polarization, as well as moderating the activity of IFNγ in the central nervous system (CNS), leading to disease improvement [46]. Consistently, a machine learning approach proposed that high DKK-3 expression related to immunosuppression was associated with poor prognosis in glioblastoma [47]. DKK-3 also affects the functions of dendritic cells (DC), which play a crucial role in initiating and regulating immune responses. Secreted DKK-3 has been shown to induce the differentiation of monocytes to DC-like cells [48]. DKK-3 is involved in inflammation, in that it reduces the intracerebral hemorrhage-related expression of TNF-α and IL-1β [25]. DKK-3 also reduces neuroinflammation and improves neuropathic pain, through the inhibition of p-ASK1, p-JNK, and p-p38, the promotion of the transformation of microglia from pro-inflammatory type M1 to anti-inflammatory type M2 and decreased production of pro-inflammatory cytokines [64].

Further research is needed to fully characterize and understand the complex interplay between DKK-3 and the immune system, and to exploit these findings for immunomodulation therapies.

## 7. DKK-3 Has a Dual Role in Cancer, as Either a Tumor Suppressor or an Oncogene

### 7.1. DKK-3 as a Tumor Suppressor

**Figure 4:**
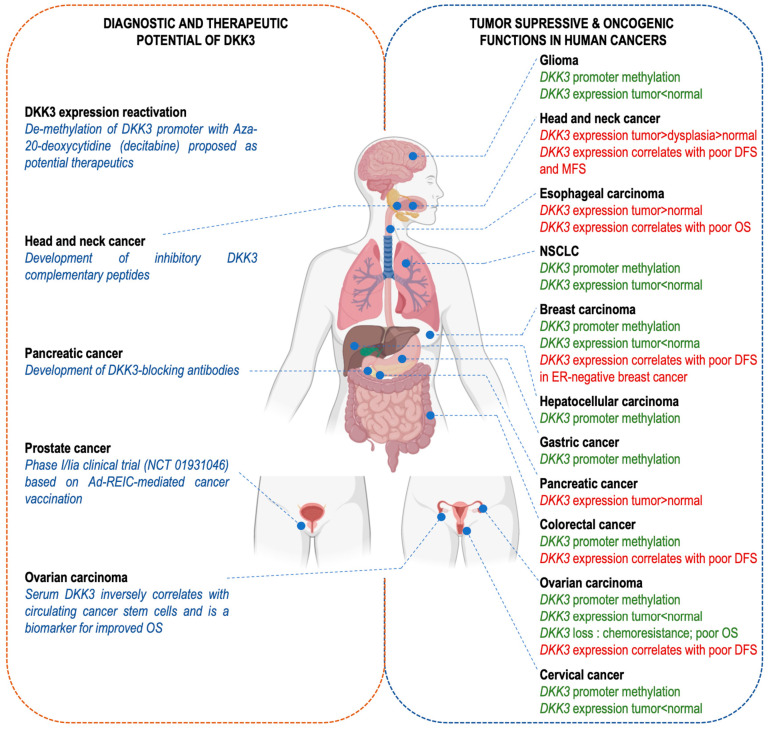
The dual role of DKK-3 in cancer: implications for diagnosis, prognosis and therapeutic strategies. DKK-3 has been reported to play a role in human cancer progression and/or response to treatment. However, its function has been described as tumor-suppressive (green text; **right** panel) or oncogenic (red text; **right** panel) depending on the cellular and tissue context. In addition, DKK-3 is a potentially interesting tool, both as a prognostic biomarker or a druggable therapeutic target (**left** panel). Created with BioRender.com (accessed on 11 November 2023).

DKK-3, as implied by its other name, REIC (reduced expression in immortalized cells), is frequently downregulated in various tumor types, cancer cell lines and immortalized cells [49], as expected for a tumor suppressor (Table 1; Figure 4). Its expression is commonly suppressed through hypermethylation of CpG islands in the DKK-3 locus in human cancer cells, including basal breast cancer, non-small cell lung cancer, gastric cancers and colon cancers [50,65]. Clinical trials are underway to explore the potential of REIC/DKK-3 gene therapy for prostate cancer [66] and liver cancer [67].

DKK-3 acts as a tumor suppressor through inhibition of the Wnt/β-catenin signaling pathway. For instance, ectopic overexpression of DKK-3 has been shown to decrease cancer cell proliferation in vitro through the inhibition of the Wnt/β-catenin pathway [51,52]. DKK-3 strongly inhibits prostate cancer cell proliferation [68]. DKK-3 expression is significantly downregulated in invasive epithelial ovarian carcinoma compared to normal tissue, adenoma, and borderline tumors. Furthermore, DKK-3 inhibits the proliferation of ovarian cancer cells. DKK-3 decreases the levels of active non-phosphorylated β-catenin, as well as P-glycoprotein, an important chemoresistance-regulating protein. DKK-3 acts as an inhibitor of chemoresistance, since reducing DKK-3 levels decreases resistance to chemotherapy with paclitaxel [53]. In lung cancer cells, DKK-3 downregulates the expression of survivin at the protein level, and DKK-3 overexpression decreases c-myc and MMP7, which are Wnt signaling effector genes that control the fate of cancer development, progression and metastasis [15]. In addition, DKK-3 inhibits cisplatin-resistant lung cancer cell growth in a xenograft model of nude mice. The authors show that DKK-3 enhances apoptosis and retards growth either alone or synergistically with cisplatin in resistant non-small cell lung cancer (NSCLC) cells (Figure 4, right panel) [15].

Interestingly, DKK-3 also exerts a tumor-suppressive function independently of Wnt signaling. An in silico study predicted an interaction between DKK-3 and EGFR, in which DKK-3 could completely occupy the EGF binding domain of the EGFR. This suggests that DKK-3 may possibly inhibit cancer proliferation by inhibiting both Wnt and EGFR signaling [24].

Another study based on blood samples from patients with ovarian cancer established a negative correlation between the serum levels of DKK-3 and the amount of circulating CD133+ cells, used as a glycoprotein marker for cancer stem cells (CSCs). Administration of DKK-3 increased both the transcript and protein levels of the epithelial marker E-cadherin and reduced mesenchymal markers (Vimentin, N-cadherin, and Snail) suggesting that DKK-3 inhibits the epithelial–mesenchymal transition (EMT). This observation is significant since it suggests the combination of both CD133+ and DKK-3 markers could have prognosis value (Figure 4, left panel) [54].

Consistently with the known function of DKK-3 as an immunomodulatory molecule (see above), REIC/DKK-3 may play a cytokine-like role in monocyte differentiation since the intratumor administration of full-length DKK-3 (FL-REIC) led to the induction of anti-cancer immunity towards prostate cancer cells, and inhibition of tumor growth in vivo [48]. Finally, our team has recently shown that DKK-3 expression is regulated by ΔNp63α in human papillomavirus-positive oropharyngeal squamous cell carcinoma, and that secreted DKK-3 activates NF-κB signaling in macrophages through a CKAP4–Akt–NF–κB axis, without impacting Wnt signaling (Figure 3B) [55].

### 7.2. DKK-3 as an Oncogene

DKK-3 expression is elevated and exhibits tumor-promoting functions in some cancers (Table 1). DKK-3 expression has been found to be high in squamous cell carcinomas of the head and neck [59] and esophagus [69], and pancreatic ductal adenocarcinoma [57], and to promote cancer cell proliferation and migration (Figure 4, right panel). Cytoplasmic levels of DKK-3 have been reported to increase during the carcinogenic transition of the oral epithelium and to be higher in tissue samples of head and neck squamous cell carcinoma (HNSCC), where they correlate with β-catenin accumulation [59]. DKK-3 stimulates processes that contribute to malignancy, which include cell-autonomous effects on cell proliferation, migration and invasion. High-level expression of DKK-3 is associated with chemoresistance and tumor volume progression in HNSCC [56,58], pancreatic [57] and esophageal cancer [10], and with poorer outcomes in HNSCC and esophageal cancer patients [10,70].

Interestingly, DKK-3 has oncogenic effects on other cells, in the tumor microenvironment, i.e., in a non-autonomous manner. DKK-3 expression has been found to be high in cancer-associated fibroblasts (CAFs) of the stroma of colon, ovarian and estrogen receptor-negative breast cancers and to be associated with worse prognosis. DKK-3 in CAFs impacts their extracellular matrix-remodeling abilities, with consequences on matrix stiffness and cancer invasion properties [26]. DKK-3 is also expressed in pancreatic stellate cells, where it promotes resistance to chemotherapy, tumor growth and metastatic spread, via both paracrine and autocrine mechanisms, through NF-κB activation [57]. Consistently with an oncogenic function, DKK-3 blockade using neutralizing antibodies increases the response to chemotherapy and the survival of mice models of pancreatic cancer [57]. High levels of stromal DKK-3 in benign prostatic hyperplasia (BPH) and prostate cancer (PCa) have been reported to increase fibroblast proliferation, promote myofibroblast differentiation, and contribute to the angiogenic switch by suppressing vessel-stabilizing factors like angiopoietin-1 (ANGPT1) (Figure 4, right panel) [60]. Finally, and as mentioned earlier, DKK-3 has an immunomodulatory role that contributes to the establishment of peripheral T-cell tolerance [46]. Thus, DKK-3 could contribute to limiting immune cell infiltration in the tumor microenvironment. Consistent with this deduction, Lu et al. showed that DKK-3 expressed by mesenchymal stem cells favors the infiltration of tumors by pro-tumorigenic M2 macrophages, and limits the recruitment of CD8+ T lymphocytes, using an immunocompetent syngeneic model of melanoma [62]. The analysis of transcriptomic data from 525 patients in The Cancer Genome Atlas database showed that DKK-3 correlates with immune suppression in glioblastoma [47,61].

In conclusion, DKK-3 appears to have a dual role, acting either to promote or repress cancer depending on the specific tissue and/or cell context. The inhibitory effect of DKK-3 on the Wnt pathway implies a potential tumor suppressor role due to the prevalent pro-oncogenic impact of Wnt pathway overactivation. However, in other tumors, DKK-3 has been implicated in immune suppression, in both Wnt signaling-dependent or independent manners.

## 8. DKK-3 as a Tool for Cancer Therapy

DKK-3 has a dual role in cancer, making its use as a diagnostic/prognostic biomarker and/or as a therapeutic target complex and context-dependent. There are various strategies to increase DKK-3 levels, where it acts as a tumor suppressor. Epigenetic reactivation of DKK-3 expression through DNA hypomethylating reagents such as 5-Aza-20-deoxycytidine (decitabine) has been proposed as a potential therapeutic [71] (Figure 4, left panel). Nevertheless, significant clinical challenges remain, such as non-selective cytotoxicity, limited bioavailability and temporary activity since DNA methylation levels revert to normal once the drug is withdrawn [72]. Attempts have been made to express DKK-3 with an adenoviral vector (Ad-REIC) in cancer patients with low levels of DKK-3. DKK-3 expression has been shown to increase cancer cell apoptosis, in a phosphorylated JNK-dependent manner, via endoplasmic reticulum (ER) stress signaling. Interestingly, in non-cancer cells, a different ER stress response is observed that leads to IL-7 expression and secretion, and NK cells activation of systemic anti-tumor immunity [73]. Ad-REIC has been shown in clinical trials (NCT01931046) with high-risk localized prostate cancer, to be safe with no apparent side effects [66] (Figure 4, left panel). However, due to the need for local administration within the tumor and the need for imaging guidance, this therapy is limited to the treatment of solid tumors.

In contrast, DKK-3 expression is upregulated in some cancers, such as squamous cell carcinoma of the head and neck and pancreatic ductal adenocarcinoma, and is correlated with poorer overall survival. DKK-3 could therefore be a druggable target and inhibitors are being developed [74,75]. Based on docking simulations, Katase et al. developed DKK-3 complementary peptides that significantly reduce DKK-3-driven Akt phosphorylation, cellular proliferation and migration of a human tongue cancer-derived cell line (HSC-3), as well as in vivo tumor growth [76]. These therapeutic peptides are a promising approach for the development of anti-cancer agents since they are relatively easy to develop and are inexpensive (Figure 4, left panel). DKK-3-blocking monoclonal antibodies have also been developed and found to be effective against pancreatic ductal adenocarcinoma progression [57] (Figure 4, left panel).

Targeting DKK-3’s oncogenic function might be challenging, due to its dual oncogenic and tumor suppressor activities (see above). Treatments that inhibit DKK-3 in tumors could inhibit tumor-suppressive functions in healthy tissues. Blocking DKK-3 could also affect Wnt/ß-catenin signaling in stem cells, where it plays an important role in cell renewal and tissue homeostasis [1]. Tumor-specific delivery could limit undesirable effects on healthy tissues. It is also conceivable that patients could have malignant clones with different susceptibility to DKK-3, in analogy with other treatments [77]. However, to the best of our knowledge, such heterogeneity has not been described.

## 9. Conclusions

DKK-3 is a target of choice for cancer therapy. There are favorable facets that suggest that DKK-3 is a target of choice. It has functions in both cancer and immune cells, suggesting that targeting one factor could have multiple and perhaps synergistic effects. Furthermore, it is secreted, which suggests that it is more easily targeted and modulated. However, to fully exploit this promise, further investigations are needed to understand the different activities of DKK-3 and to optimize therapeutic strategies that take into account context and cancer-type dependence.

## Figures and Tables

**Table 1:** A comprehensive literature summary: revealing the versatile role of human Dickkopf-3 (DKK-3) in development, immune modulation, and cancer.

	DKK-3 Function	Comment	Ref.
**Cell protection**	Cell adhesion, migration and invasion	Secreted DKK-3 is part of the extracellular matrix and promotes cell adhesion, migration and invasion	[38]
	Chondroprotection	DKK-3 prevents interleukin-1β/oncostatin-M driven proteoglycan loss of cartilage and is upregulated in osteoarthritis	[39]
	Protection against cardiac hypertrophy	DKK-3 protects the heart against pressure overload-induced cardiac remodeling through the inhibition of the ASK1-JNK/p38 signaling pathway	[40]
	Smooth muscle cell (SMC) differentiation	DKK-3 induces the expression of differentiated SMC markers (myocardin) through the activation of the ATF6 transcription factor	[41]
	Endothelium protection and repair	*DKK-3^−/−^* mice have more atherosclerotic lesions. DKK-3 stimulates endothelial cell migration through the Wnt/planar cell polarity signaling pathway	[42]
	Protection against neuronal death	*DKK-3^−/−^* ischemic mice have greater infarct size. DKK-3 prevents astrocyte death via the upregulation of VEGF expression and the activation of VEGFR2	[43]
**Immune response and inflammation**	B-cell fate and function	B1 cells have better proliferation and survival abilities whereas the development of B2 cells is impaired in *DKK-3^−/−^* mice, which also show altered immune and cytokine responses	[44]
	Peripheral CD8 T-cell tolerance	DKK-3 is expressed in tolerant T-cells, where it reduces reactivity to host antigens. Blocking DKK-3 leads to autologous skin graft rejection. *DKK-3^−/−^* mice suffer from exacerbated experimental autoimmune encephalomyelitis with increased numbers of IFNγ-producing T-cells in the central nervous system	[45,46]
	Anti-tumor immune response regulation	High expression of DKK-3 in glioblastoma multiform correlates with poorer prognosis and decreased anti-tumor immunity.	[47]
	Regulation of inflammatory cytokines	DKK-3 reduces the intracerebral hemorrhage-related expression of TNF-α and IL-1β	[25]
	Differentiation of monocytes to DCs	Human CD14+ monocytes grown with recombinant DKK-3 differentiate into immature CD11c+, CD40+, CD86+, HLADR+ DCs	[48]
	Microglia polarization and reduction of neuroinflammation	DKK-3 inhibits the ASK-1/JNK/p38 pathway, favoring the transformation of type M1 to M2 microglia and the downregulation of TNF-α, IL-6 and IL-1β	[25]
**Tumor suppressor function**	Downregulated expression in cancer cells	DKK-3 expression is lower in immortalized cell lines and 8 human tumor-derived cell lines	[49]
	DKK-3 promoter is hypermethylated in cancer	The frequency of *DKK-3* promoter hypermethylation varies from 26% (pleural mesothelia) to 61% (breast carcinoma).	[7,50]
	Suppression of cell proliferation	The overexpression of DKK-3 in hepatoma and cervical cancer cells diminishes cell proliferation in vitro	[51,52]
	Response to chemotherapy	DKK-3 loss is correlated with chemoresistance and adverse prognosis in ovarian cancer, and extracellular DKK-3 reduces cell proliferation, migration, and response to paclitaxel in vitro *DKK-3* siRNA-mediated downregulation mediates lung cancer cell resistance to cisplatin, and DKK-3 overexpression impairs cell proliferation and migration, induces apoptosis and increases cell sensitivity to cisplatin.	[15,53]
	Serum DKK-3 is a prognosis biomarker	DKK-3 levels are negatively correlated with the number of CD133+ circulating tumor cells in ovarian cancer	[54]
	Regulation of the tumor immune microenvironment	DKK-3 expression correlates with infiltrating CD8+ lymphocytes and macrophages in HPV-positive HNSCC and stimulates NF-κB signaling in macrophages	[55]
**Oncogenic function**	Stimulation of cell proliferation, migration and tumor growth	The expression of DKK-3 is high in HNSCC, esophageal and pancreatic cell lines, where it stimulates cell proliferation and migration in vitro. In xenograft models, DKK-3 supports tumor growth.	[10,56,57,58]
	Correlation with tumor progression	DKK-3 expression was found to increase from normal oral mucosa in dysplasia and squamous cell carcinoma and to correlate with the tissue proliferative index (Ki67)	[59]
	DKK-3 has pro-tumorigenic function in tumor stroma	The expression of DKK-3 in the stroma of breast, colon and ovarian cancer is associated with poorer prognosis. DKK-3 stimulates the β-catenin and YAP/TAZ signaling pathways in cancer-associated fibroblasts, increasing their extracellular matrix remodeling activity. The knockdown of DKK-3 in prostatic stromal cells decreases their proliferation, inhibits the fibroblast-to-myofibroblast differentiation, and contributes to blood vessel destabilization through the upregulation of ANGPT1.	[26,60]
	DKK-3 impairs anti-cancer immunity	DKK-3 expression by mesenchymal stem cells favors the infiltration of tumors by pro-tumorigenic M2 macrophages and limits the recruitment of CD8+ T lymphocytes. DKK-3 expression correlates with immune suppression in glioblastoma.	[29,30,31,47,61,62]

## Data Availability

Not applicable.

## References

[1] Mannino G., Russo C., Maugeri G., Musumeci G., Vicario N., Tibullo D., Giuffrida R., Parenti R., Lo Furno D. (2022). Adult Stem Cell Niches for Tissue Homeostasis. J. Cell Physiol..

[2] Aiello N.M., Stanger B.Z. (2016). Echoes of the Embryo: Using the Developmental Biology Toolkit to Study Cancer. Dis. Models Mech..

[3] Zhang Y., Wang X. (2020). Targeting the Wnt/β-Catenin Signaling Pathway in Cancer. J. Hematol. Oncol..

[4] Kikuchi A., Matsumoto S., Sada R. (2022). Dickkopf Signaling, beyond Wnt-Mediated Biology. Semin. Cell Dev. Biol..

[5] Giralt I., Gallo-Oller G., Navarro N., Zarzosa P., Pons G., Magdaleno A., Segura M.F., Sánchez de Toledo J., Moreno L., Gallego S. (2021). Dickkopf Proteins and Their Role in Cancer: A Family of Wnt Antagonists with a Dual Role. Pharmaceuticals.

[6] Kaneko K.J., DePamphilis M.L. (2000). Soggy, a Spermatocyte-Specific Gene, Lies 3.8 Kb Upstream of and Antipodal to TEAD-2, a Transcription Factor Expressed at the Beginning of Mouse Development. Nucleic Acids Res..

[7] Veeck J., Dahl E. (2012). Targeting the Wnt Pathway in Cancer: The Emerging Role of Dickkopf-3. Biochim. Et Biophys. Acta (BBA)-Rev. Cancer.

[8] Krupnik V.E., Sharp J.D., Jiang C., Robison K., Chickering T.W., Amaravadi L., Brown D.E., Guyot D., Mays G., Leiby K. (1999). Functional and Structural Diversity of the Human Dickkopf Gene Family. Gene.

[9] Niehrs C. (2006). Function and Biological Roles of the Dickkopf Family of Wnt Modulators. Oncogene.

[10] Kajiwara C., Fumoto K., Kimura H., Nojima S., Asano K., Odagiri K., Yamasaki M., Hikita H., Takehara T., Doki Y. (2018). P63-Dependent Dickkopf3 Expression Promotes Esophageal Cancer Cell Proliferation via CKAP4. Cancer Res..

[11] Kawano Y., Kypta R. (2003). Secreted Antagonists of the Wnt Signalling Pathway. J. Cell Sci..

[12] Hayat R., Manzoor M., Hussain A. (2022). Wnt Signaling Pathway: A Comprehensive Review. Cell Biol. Int..

[13] Aravind L., Koonin E.V. (1998). A Colipase Fold in the Carboxy-Terminal Domain of the Wnt Antagonists—The Dickkopfs. Curr. Biol..

[14] Kohn A.D., Moon R.T. (2005). Wnt and Calcium Signaling: Beta-Catenin-Independent Pathways. Cell Calcium..

[15] Wang Z., Ma L.-J., Kang Y., Li X., Zhang X.-J. (2015). Dickkopf-3 (Dkk3) Induces Apoptosis in Cisplatin-Resistant Lung Adenocarcinoma Cells via the Wnt/β-Catenin Pathway. Oncol. Rep..

[16] Duchartre Y., Kim Y.-M., Kahn M. (2016). The Wnt Signaling Pathway in Cancer. Crit. Rev. Oncol. Hematol..

[17] Schunk S.J., Floege J., Fliser D., Speer T. (2021). WNT–β-catenin signalling—A versatile player in kidney injury and repair. Nat. Rev. Nephrol..

[18] MacDonald B.T., He X. (2012). Frizzled and LRP5/6 Receptors for Wnt/β-Catenin Signaling. Cold Spring Harb. Perspect. Biol..

[19] Mao B., Wu W., Li Y., Hoppe D., Stannek P., Glinka A., Niehrs C. (2001). LDL-Receptor-Related Protein 6 Is a Receptor for Dickkopf Proteins. Nature.

[20] Mao B., Wu W., Davidson G., Marhold J., Li M., Mechler B.M., Delius H., Hoppe D., Stannek P., Walter C. (2002). Kremen Proteins Are Dickkopf Receptors That Regulate Wnt/b-Catenin Signalling. Nature.

[21] Nakamura T., Aoki S., Kitajima K., Takahashi T., Matsumoto K., Nakamura T. (2001). Molecular Cloning and Characterization of Kremen, a Novel Kringle-Containing Transmembrane Protein. Biochim. Et Biophys. Acta (BBA)-Gene Struct. Expr..

[22] Mao B., Niehrs C. (2003). Kremen2 Modulates Dickkopf2 Activity during Wnt/lRP6 Signaling. Gene.

[23] Mohammadpour H., Khalili S., Hashemi Z.S. (2015). Kremen Is beyond a Subsidiary Co-Receptor of Wnt Signaling: An in Silico Validation. Turk. J. Biol..

[24] Mohammadpour H., Pourfathollah A.A., Nikougoftar Zarif M., Khalili S. (2016). Key Role of Dkk3 Protein in Inhibition of Cancer Cell Proliferation: An In Silico Identification. J. Theor. Biol..

[25] Xu Y., Nowrangi D., Liang H., Wang T., Yu L., Lu T., Lu Z., Zhang J.H., Luo B., Tang J. (2020). DKK3 Attenuates JNK and AP-1 Induced Inflammation via Kremen-1 and DVL-1 in Mice Following Intracerebral Hemorrhage. J. Neuroinflamm..

[26] Ferrari N., Ranftl R., Chicherova I., Slaven N.D., Moeendarbary E., Farrugia A.J., Lam M., Semiannikova M., Westergaard M.C.W., Tchou J. (2019). Dickkopf-3 Links HSF1 and YAP/TAZ Signalling to Control Aggressive Behaviours in Cancer-Associated Fibroblasts. Nat. Commun..

[27] Leonard D.M., Stachelek S.J., Safran M., Farwell A.P., Kowalik T.F., Leonard J.L. (2000). Cloning, Expression, and Functional Characterization of the Substrate Binding Subunit of Rat Type II Iodothyronine 5′-Deiodinase. J. Biol. Chem..

[28] Stachelek S.J., Tuft R.A., Lifschitz L.M., Leonard D.M., Farwell A.P., Leonard J.L. (2001). Real-Time Visualization of Processive Myosin 5a-Mediated Vesicle Movement in Living Astrocytes * 210. J. Biol. Chem..

[29] Katase N., Nagano K., Fujita S. (2020). DKK3 Expression and Function in Head and Neck Squamous Cell Carcinoma and Other Cancers. J. Oral Biosci..

[30] Leonard J.L., Leonard D.M., Wolfe S.A., Liu J., Rivera J., Yang M., Leonard R.T., Johnson J.P.S., Kumar P., Liebmann K.L. (2017). The Dkk3 Gene Encodes a Vital Intracellular Regulator of Cell Proliferation. PLoS ONE.

[31] Kimura H., Fumoto K., Shojima K., Nojima S., Osugi Y., Tomihara H., Eguchi H., Shintani Y., Endo H., Inoue M. (2016). CKAP4 Is a Dickkopf1 Receptor and Is Involved in Tumor Progression. J. Clin. Investig..

[32] Sandoz P.A., Denhardt-Eriksson R.A., Abrami L., Abriata L.A., Spreemann G., Maclachlan C., Ho S., Kunz B., Hess K., Knott G. (2023). Dynamics of CLIMP-63 S-Acylation Control ER Morphology. Nat. Commun..

[33] Kikuchi A., Fumoto K., Kimura H. (2017). The Dickkopf1-Cytoskeleton-Associated Protein 4 Axis Creates a Novel Signalling Pathway and May Represent a Molecular Target for Cancer Therapy. Br. J. Pharmacol..

[34] Bhavanasi D., Speer K.F., Klein P.S. (2016). CKAP4 Is Identified as a Receptor for Dickkopf in Cancer Cells. J. Clin. Investig..

[35] del Barrantes I.B., Montero-Pedrazuela A., Guadaño-Ferraz A., Obregon M.-J., Martinez de Mena R., Gailus-Durner V., Fuchs H., Franz T.J., Kalaydjiev S., Klempt M. (2006). Generation and Characterization of Dickkopf3 Mutant Mice. Mol. Cell Biol..

[36] Bertozzi A., Wu C.-C., Hans S., Brand M., Weidinger G. (2022). Wnt/β-Catenin Signaling Acts Cell-Autonomously to Promote Cardiomyocyte Regeneration in the Zebrafish Heart. Dev. Biol..

[37] Singh S.P., Chawla P., Hnatiuk A., Kamel M., Silva L.D., Spanjaard B., Eski S.E., Janjuha S., Olivares-Chauvet P., Kayisoglu O. (2022). A Single-Cell Atlas of de Novo β-Cell Regeneration Reveals the Contribution of Hybrid β/δ-Cells to Diabetes Recovery in Zebrafish. Development.

[38] Kano J., Wang H., Zhang H., Noguchi M. (2022). Roles of DKK3 in Cellular Adhesion, Motility, and Invasion through Extracellular Interaction with TGFBI. FEBS J..

[39] Snelling S.J.B., Davidson R.K., Swingler T.E., Le L.T.T., Barter M.J., Culley K.L., Price A., Carr A.J., Clark I.M. (2016). Dickkopf-3 Is Upregulated in Osteoarthritis and Has a Chondroprotective Role. Osteoarthr. Cartil..

[40] Zhang Y., Liu Y., Zhu X.-H., Zhang X.-D., Jiang D.-S., Bian Z.-Y., Zhang X.-F., Chen K., Wei X., Gao L. (2014). Dickkopf-3 Attenuates Pressure Overload-Induced Cardiac Remodelling. Cardiovasc. Res..

[41] Wang X., Karamariti E., Simpson R., Wang W., Xu Q. (2015). Dickkopf Homolog 3 Induces Stem Cell Differentiation into Smooth Muscle Lineage via ATF6 Signalling. J. Biol. Chem..

[42] Yu B., Kiechl S., Qi D., Wang X., Song Y., Weger S., Mayr A., Le Bras A., Karamariti E., Zhang Z. (2017). A Cytokine-like Protein Dickkopf-Related Protein 3 Is Atheroprotective. Circulation.

[43] Busceti C.L., Di Menna L., Bianchi F., Mastroiacovo F., Di Pietro P., Traficante A., Bozza G., Niehrs C., Battaglia G., Bruno V. (2018). Dickkopf-3 Causes Neuroprotection by Inducing Vascular Endothelial Growth Factor. Front. Cell Neurosci..

[44] Ludwig J., Federico G., Prokosch S., Küblbeck G., Schmitt S., Klevenz A., Gröne H.-J., Nitschke L., Arnold B. (2015). Dickkopf-3 Acts as a Modulator of B Cell Fate and Function. J. Immunol..

[45] Papatriantafyllou M., Moldenhauer G., Ludwig J., Tafuri A., Garbi N., Hollmann G., Küblbeck G., Klevenz A., Schmitt S., Pougialis G. (2012). Dickkopf-3, an Immune Modulator in Peripheral CD8 T-Cell Tolerance. Proc. Natl. Acad. Sci. USA.

[46] Meister M., Papatriantafyllou M., Nordström V., Kumar V., Ludwig J., Lui K.O., Boyd A.S., Popovic Z.V., Fleming T.H., Moldenhauer G. (2015). Dickkopf-3, a Tissue-Derived Modulator of Local T-Cell Responses. Front. Immunol..

[47] Han M.-H., Min K.-W., Noh Y.-K., Kim J.M., Cheong J.H., Ryu J.I., Won Y.D., Koh S.-H., Myung J.K., Park J.Y. (2022). High DKK3 Expression Related to Immunosuppression Was Associated with Poor Prognosis in Glioblastoma: Machine Learning Approach. Cancer Immunol. Immunother..

[48] Watanabe M., Kashiwakura Y., Huang P., Ochiai K., Futami J., Li S.-A., Takaoka M., Nasu Y., Sakaguchi M., Huh N.-H. (2009). Immunological Aspects of REIC/Dkk-3 in Monocyte Differentiation and Tumor Regression. Int. J. Oncol..

[49] Tsuji T., Miyazaki M., Sakaguchi M., Inoue Y., Namba M. (2000). A REIC Gene Shows Down-Regulation in Human Immortalized Cells and Human Tumor-Derived Cell Lines. Biochem. Biophys. Res. Commun..

[50] Hayashi T., Asano H., Toyooka S., Tsukuda K., Soh J., Shien T., Taira N., Maki Y., Tanaka N., Doihara H. (2012). DNA Methylation Status of REIC/Dkk-3 Gene in Human Malignancies. J. Cancer Res. Clin. Oncol..

[51] Hsieh S.-Y., Hsieh P.-S., Chiu C.-T., Chen W.-Y. (2004). Dickkopf-3/REIC Functions as a Suppressor Gene of Tumor Growth. Oncogene.

[52] Lee E.-J., Jo M., Rho S.B., Park K., Yoo Y.-N., Park J., Chae M., Zhang W., Lee J.-H. (2009). Dkk3, Downregulated in Cervical Cancer, Functions as a Negative Regulator of β-Catenin. Int. J. Cancer.

[53] Nguyen Q.T.T., Park H.S., Lee T.J., Choi K.-M., Park J.Y., Kim D., Kim J.H., Park J., Lee E.-J. (2022). DKK3, Downregulated in Invasive Epithelial Ovarian Cancer, Is Associated with Chemoresistance and Enhanced Paclitaxel Susceptibility via Inhibition of the β-Catenin-P-Glycoprotein Signaling Pathway. Cancers.

[54] Nie X.-C., He F., Lan C., Niu J.-M., Xia P. (2021). Combined Serum DKK3 and Circulating CD133 Cells as Prognostic Biomarkers for Ovarian Cancer Patients. OncoTargets Ther..

[55] Mourtada J., Lony C., Nicol A., De Azevedo J., Bour C., Macabre C., Roncarati P., Ledrappier S., Schultz P., Borel C. (2023). A Novel ΔNp63-Dependent Immune Mechanism Improves Prognosis of HPV-Related Head and Neck Cancer. Front. Immunol..

[56] Katase N., Nishimatsu S.-I., Yamauchi A., Yamamura M., Terada K., Itadani M., Okada N., Hassan N.M.M., Nagatsuka H., Ikeda T. (2018). DKK3 Overexpression Increases the Malignant Properties of Head and Neck Squamous Cell Carcinoma Cells. Oncol. Res..

[57] Zhou L., Husted H., Moore T., Lu M., Deng D., Liu Y., Ramachandran V., Arumugam T., Niehrs C., Wang H. (2018). Suppression of Stromal-Derived Dickkopf-3 (DKK3) Inhibits Tumor Progression and Prolongs Survival in Pancreatic Ductal Adenocarcinoma. Sci. Transl. Med..

[58] Katase N., Nishimatsu S.-I., Yamauchi A., Yamamura M., Fujita S. (2019). DKK3 Knockdown Confers Negative Effects on the Malignant Potency of Head and Neck Squamous Cell Carcinoma Cells via the PI3K/Akt and MAPK Signaling Pathways. Int. J. Oncol..

[59] Fujii M., Katase N., Lefeuvre M., Gunduz M., Buery R.R., Tamamura R., Tsujigiwa H., Nagatsuka H. (2011). Dickkopf (Dkk)-3 and β-Catenin Expressions Increased in the Transition from Normal Oral Mucosal to Oral Squamous Cell Carcinoma. J. Mol. Hist..

[60] Zenzmaier C., Sampson N., Plas E., Berger P. (2013). Dickkopf-related Protein 3 Promotes Pathogenic Stromal Remodeling in Benign Prostatic Hyperplasia and Prostate Cancer. Prostate.

[61] Han M.-H., Baek J.M., Min K.-W., Cheong J.H., Ryu J.I., Won Y.D., Kwon M.J., Koh S.-H. (2023). DKK3 Expression Is Associated with Immunosuppression and Poor Prognosis in Glioblastoma, in Contrast to Lower-Grade Gliomas. BMC Neurol..

[62] Lu K.-H., Tounsi A., Shridhar N., Küblbeck G., Klevenz A., Prokosch S., Bald T., Tüting T., Arnold B. (2015). Dickkopf-3 Contributes to the Regulation of Anti-Tumor Immune Responses by Mesenchymal Stem Cells. Front. Immunol..

[63] Akazawa H., Komuro I. (2014). Dickkopf-3: A Stubborn Protector of Cardiac Hypertrophy. Cardiovasc. Res..

[64] Zhang L.-Q., Gao S.-J., Sun J., Li D.-Y., Wu J.-Y., Song F.-H., Liu D.-Q., Zhou Y.-Q., Mei W. (2022). DKK3 Ameliorates Neuropathic Pain via Inhibiting ASK-1/JNK/p-38-Mediated Microglia Polarization and Neuroinflammation. J. Neuroinflamm..

[65] Veeck J., Bektas N., Hartmann A., Kristiansen G., Heindrichs U., Knüchel R., Dahl E. (2008). Wnt Signalling in Human Breast Cancer: Expression of the Putative Wnt Inhibitor Dickkopf-3 (DKK3) Is Frequently Suppressed by Promoter Hypermethylation in Mammary Tumours. Breast Cancer Res..

[66] Kumon H., Ariyoshi Y., Sasaki K., Sadahira T., Araki M., Ebara S., Yanai H., Watanabe M., Nasu Y. (2016). Adenovirus Vector Carrying REIC/DKK-3 Gene: Neoadjuvant Intraprostatic Injection for High-Risk Localized Prostate Cancer Undergoing Radical Prostatectomy. Cancer Gene Ther..

[67] Oyama A., Shiraha H., Uchida D., Iwamuro M., Kato H., Takaki A., Ikeda F., Onishi H., Yasunaka T., Takeuchi Y. (2019). A Phase I/Ib Trial of Ad-REIC in Liver Cancer: Study Protocol. Future Oncol..

[68] Romero D., Kypta R. (2013). Dickkopf-3 Function in the Prostate. Bioarchitecture.

[69] Wang Z., Lin L., Thomas D.G., Nadal E., Chang A.C., Beer D.G., Lin J. (2015). The Role of Dickkopf-3 Overexpression in Esophageal Adenocarcinoma. J. Thorac. Cardiovasc. Surg..

[70] Katase N., Nishimatsu S.-I., Yamauchi A., Okano S., Fujita S. (2023). DKK3 Expression Is Correlated with Poorer Prognosis in Head and Neck Squamous Cell Carcinoma: A Bioinformatics Study Based on the TCGA Database. J. Oral. Biosci..

[71] Hamzehzadeh L., Caraglia M., Atkin S.L., Sahebkar A. (2018). Dickkopf Homolog 3 (DKK3): A Candidate for Detection and Treatment of Cancers?. J. Cell. Physiol..

[72] Mehdipour P., Murphy T., De Carvalho D.D. (2020). The Role of DNA-Demethylating Agents in Cancer Therapy. Pharmacol. Ther..

[73] Watanabe M., Nasu Y., Kumon H. (2014). Adenovirus-Mediated REIC/Dkk-3 Gene Therapy: Development of an Autologous Cancer Vaccination Therapy (Review). Oncol. Lett..

[74] Katase N., Lefeuvre M., Gunduz M., Gunduz E., Beder L.B., Grenman R., Fujii M., Tamamu R., Tsujigiwa H., Nagatsuka H. (2012). Absence of Dickkopf (Dkk)-3 Protein Expression Is Correlated with Longer Disease-Free Survival and Lower Incidence of Metastasis in Head and Neck Squamous Cell Carcinoma. Oncol. Lett..

[75] Zenzmaier C., Hermann M., Hengster P., Berger P. (2012). Dickkopf-3 Maintains the PANC-1 Human Pancreatic Tumor Cells in a Dedifferentiated State. Int. J. Oncol..

[76] Katase N., Nishimatsu S., Yamauchi A., Okano S., Fujita S. (2022). Establishment of Anti-DKK3 Peptide for the Cancer Control in Head and Neck Squamous Cell Carcinoma (HNSCC). Cancer Cell Int..

[77] Marusyk A., Janiszewska M., Polyak K. (2020). Intratumor Heterogeneity: The Rosetta Stone of Therapy Resistance. Cancer. Cell.

